# Synthesis and Functional Characterization of Substituted Isoquinolinones as MT_2_-Selective Melatoninergic Ligands

**DOI:** 10.1371/journal.pone.0113638

**Published:** 2014-12-05

**Authors:** Yueqing Hu, King H. Chan, Xixin He, Maurice K. C. Ho, Yung H. Wong

**Affiliations:** 1 Division of Life Science and the Biotechnology Research Institute, Hong Kong University of Science and Technology, Clear Water Bay, Kowloon, Hong Kong; 2 State Key Laboratory of Molecular Neuroscience, and the Molecular Neuroscience Center, Hong Kong University of Science and Technology, Clear Water Bay, Kowloon, Hong Kong; University of Parma, Italy

## Abstract

A series of substituted isoquinolinones were synthesized and their binding affinities and functional activities towards human melatonin MT_1_ and MT_2_ receptors were evaluated. Structure-activity relationship analysis revealed that substituted isoquinolinones bearing a 3-methoxybenzyloxyl group at C5, C6 or C7 position respectively (C5>C6>C7 in terms of their potency) conferred effective binding and selectivity toward the MT_2_ receptor, with **15b** as the most potent compound. Most of the tested compounds were MT_2_-selective agonists as revealed in receptor-mediated cAMP inhibition, intracellular Ca^2+^ mobilization and phosphorylation of extracellular signal-regulated protein kinases. Intriguingly, compounds **7e** and **7f** bearing a 4-methoxybenzyloxyl group or 4-methylbenzyloxyl at C6 behaved as weak MT_2_-selective antagonists. These results suggest that substituted isoquinolinones represent a novel family of MT_2_-selective melatonin ligands. The position of the substituted benzyloxyl group, and the substituents on the benzyl ring appeared to dictate the functional characteristics of these compounds.

## Introduction

Melatonin (N-acetyl-5-methoxytryptamine) is a versatile hormone which regulates circadian rhythm as well as many other biological functions [1]–[3]. It is secreted by the pineal gland and its biological effects are exerted through specific melatonin binding sites. Two of them belong to the family of seven-transmembrane-domain G protein-coupled receptors (GPCR) have been cloned (MT_1_ and MT_2_), and shown to be expressed in mammals [4]–[6]. They are believed to play different, or in some circumstances opposite, roles in biological systems [7]. MT_1_ receptors modulate neuronal firing, arterial vasoconstriction, cell proliferation, reproductive, and metabolic functions [8]–[12]. MT_2_ receptors are responsible for resetting the circadian rhythm of neuronal firing in the suprachiasmatic nucleus (phase-shifting), inhibiting dopamine release in retina, inducing vasodilation, and enhancing immune responses [9], [13]–[15]. While the melatonin receptor subtypes may work in concert to regulate various chronobiotic and homeostatic responses, the distinct roles of MT_1_ and MT_2_ spur the interest to develop subtype-specific pharmacological agents to pinpoint their individual roles in the regulation of circadian rhythmicity, or promoting sleep without phase-shifting the circadian clock.

The therapeutic potential of melatonin is limited by its non-subtype specific actions at multiple receptors as well as its unfavorable pharmacokinetic properties, such as high first-pass metabolism, short half-life and poor oral bioavailability [16], [17]. Novel melatoninergic compounds with different chemical scaffolds have therefore been synthesized and discovered, such as indoles, naphthlenes, benzoxazoles, pyrrolidines, and tetralins. Many of the compounds consist of an alkylamide with a terminal alkyl chain not longer than 3–4 carbons, which mainly governs the binding affinity towards the melatonin receptors. A number of early studies have also shown that the presence of an aromatic substituent on the C2 position of the melatonin indole ring can confer MT_2_ selectivity [18], [19], but none of these selective melatoninergic agents had been developed into clinical uses. To date, the area of subtype-selective therapeutic melatoninergics has not been thoroughly addressed.

Our research goals were to discover novel compounds that exhibit potent binding affinity and good subtype selectivity at MT_1_ and/or MT_2_ receptors. The compound 7-hydroxy-6-methoxy-2-methyl-2*H*-isoquinolin-1-one (compound **12**) was identified as a modest melatoninergic agonist with selectivity towards MT_2_ in high-throughput drug screening assays (Figure S1). An obvious distinctive feature of this compound as compared to melatonin and many other melatoninergics is the lack of free alkylamide side chain but a *N*-methylamide moiety confined in the isoquinolinone scaffold, and there is no previous evidence showing any structural resemblance or functional equivalence of such *N*-methylamide moiety to the alkylamide chain of other known melatoninergics.

As isoquinolinone represents a novel chemical scaffold possessing melatoninergic activity, it prompted us to develop a series of more potent and selective isoquinolinone derivatives. In this study, a series of substituted isoquinolinones were synthesized. Different substituted benzyloxyl and methoxy substituents were incorporated at C5–C7 positions of the isoquinolinone ring to generate sufficient derivatives for structure-activity relationship analysis. The binding affinities of the tested compounds were evaluated in competitive receptor binding assays using radiolabeled melatonin as the probe on intact cells expressing each of the recombinant melatonin receptor subtypes. The abilities of the tested compounds to trigger receptor-mediated inhibition of cAMP production, intracellular Ca^2+^ mobilization and phosphorylation of extracellular signal-regulated protein kinases (ERK) were compared with melatonin-induced responses.

## Chemistry

**Figure 1:**
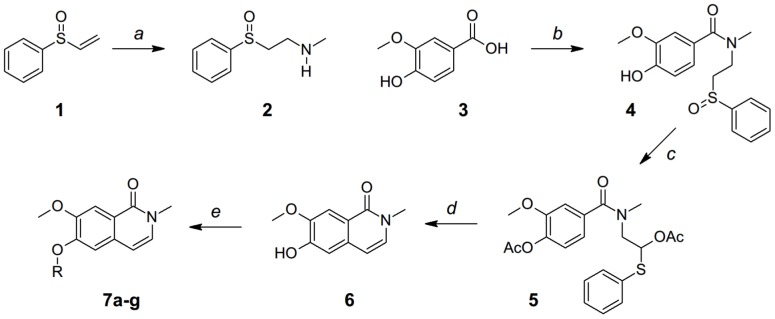
Synthesis of isoquinolinones 7a–g. a) MeNH_2_, THF, r.t., ∼75%; b) DIC, **2**, CH_2_Cl_2_, r.t., 84–91%; c) Ac_2_O, reflux, ∼100%; d) TsOH, toluene, heat, 71% for two steps; e) RX, K_2_CO_3_, DMF, r.t., ∼95%.

**Figure 2:**
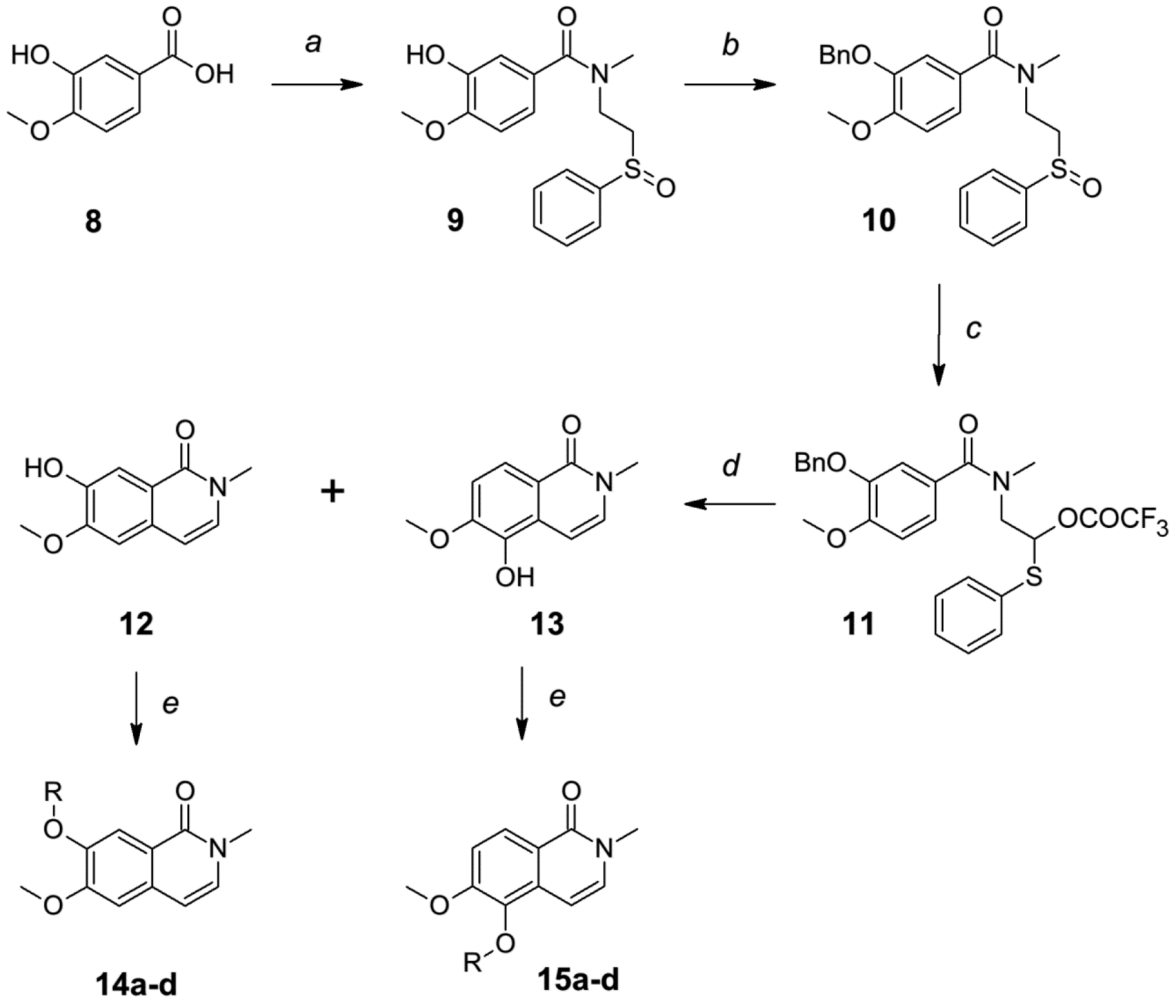
Synthesis of isoquinolinones 14a–d and 15a–d. a) DIC, **2**, CH_2_Cl_2_, r.t. 84–91%; b) BnBr, K_2_CO_3_, DMF, 91% for two steps; c) TFAA, CH_2_Cl_2_, 0°C; d) TsOH, toluene, heat, yields for two steps: 37% for **12**, 24% for **13**. e) RX, K_2_CO_3_, DMF, r.t., ∼95%.

Isoquinolinone compounds were synthesized in 4–5 steps, using Lee's method [20] (Figure 1) or its modified version (Figure 2). Starting with commercially available phenylvinyl sulfoxide **1**, 1,4-conjugate addition with methylamine formed the amine **2**. Coupling of 4-hydroxy-3-methoxybenzoic acid **3** with amine **2** generated the amide **4**. Pummerer rearrangement [21], [22] of **4** by refluxing with acetic anhydride converted the sulfoxide to masked “acetal” compound **5**. Heating compound **5** in toluene under acidic conditions (*p*-toluenesulfonic acid, TsOH) completed the heterocyclic ring to give isoquinolinone **6** in 71% yield together with minor *O*-methyl migrated byproduct. Alkylation of the free hydroxyl group of **6** with different halides yielded a series of isoquinolinones **7a–7g** with different substituents at C6 position.

For analogues with different substitutions at C7 (compounds **14a–d**) or C5 (compounds **15a–d**) positions, 3-hydroxy-4-methoxybenzoic acid **8** was used as the starting material. The Lee's method was initially attempted. Coupling of acid **8** with amine **2** formed the amide **9**. Pummerer rearrangement of amide **9** in refluxing acetic anhydride followed by acidic cyclization provided a complex of products resulting from clockwise and anti-clockwise cyclizations and *O*-methyl group migrations. In order to simplify the product isolation, the hydroxyl group of compound **9** was protected as benzyl ether **10**. Refluxing of **10** in acetic anhydride followed by acidic cyclization provided a similar complex of products without the benzyl protection group. The Pummerer rearrangement reaction was performed with trifluoroacetic anhydride (TFAA) in dichloromethane at 0°C. After acidic cyclization, isoquinolinones **12** and **13** were produced with satisfactory yields with low amount of *O*-methyl group migration byproducts. Alkylations of **12** or **13** with different halides gave the desired analogues **14a–d** or **15a–d**, respectively.

## Results

### Competitive binding assays

Table 1 shows the compound structures and their binding affinities toward human MT_1_ and MT_2_ receptors. Competitive receptor binding characteristics of melatonin and the 15 tested compounds towards human MT_1_ and MT_2_ stably expressed in Chinese hamster ovary (CHO) cells were determined using intact cell preparation pre-incubated with 1 nM [^3^H]melatonin. The K_d_ of melatonin for MT_1_ and MT_2_ receptors was 0.30 nM and 0.43 nM, respectively, as determined by saturation binding assays. All of the tested compounds showed very weak (K_i_>1 µM) or basically no significant binding towards MT_1_ (Table 1). Contrarily, most of the tested compounds showed detectable binding at MT_2_. Among compounds **7a**–**7g** with a benzyloxyl or substituted benzyloxyl group at C6, **7b** which bears a 3-methoxybenzyloxyl group displayed the most outstanding binding affinity, consistent with our previous report [23]. **7b** was also the only compound among this group which completely displaced the pre-incubated [^3^H]melatonin.

**Table 1:** Binding affinities of the tested compounds towards human MT_1_ and MT_2_ expressed in CHO cells.

		MT_1_			MT_2_			
Compound	R	pIC_50_±SEM	K_i_ (nM)	%Resp^a^±SEM	pIC_50_±SEM	K_i_ (nM)	%Resp±SEM	Selectivity^b^
MT	Bn	9.53±0.14	0.296	95±2	9.37±0.21	0.429	91±6	0.69
**7a**	Bn	NSB^c^	-	-	5.55±0.18	852	78±7	-
**7b**	(3-MeO)Bn	4.79±0.14	3700	90±8	7.05±0.16	26.6	92±4	139
**7c**	(3,5-MeO)Bn	4.24±0.24	13100	41±9	5.93±0.54	350	73±9	37.6
**7d**	(2-MeO)Bn	NSB	-	-	5.65±0.17	674	79±5	-
**7e**	(4-MeO)Bn	NSB	-	-	5.95±0.34	334	47±5	-
**7f**	(4-Me)Bn	5.05±0.06	2040	38±10	5.96±0.40	333	70±6	6.13
**7g**	(3,5-Me)Bn	NSB	-	-	5.46±0.27	1050	86±2	-
**14a**	Bn	NSB	-	-	4.83±0.24	4460	78±8	-
**14b**	(3-MeO)Bn	NSB	-	-	6.00±0.24	302	92±3	-
**14c**	(3,5-MeO)Bn	NSB	-	-	4.74±0.60	5490	94±11	-
**14d**	(4-Me)Bn	NSB	-	-	4.86±0.15	4150	73±10	-
**15a**	Bn	5.04±0.42	2070	84±10	5.92±0.37	362	85±8	5.72
**15b**	(3-MeO)Bn	4.88±0.15	2980	71±13	8.26±0.06	1.66	85±8	1800
**15c**	(3,5-MeO)Bn	5.03±0.12	2130	63±4	6.85±0.16	42.0	94±13	50.7
**15d**	(4-Me)Bn	5.47±0.35	770	34±5	5.02±0.38	2890	74±11	0.27

a %Resp – Percentage responses of the melatonin (MT)-mediated displacement of radioligand binding.

b Selectivity was defined as the ratio K_i_(MT_1_)/K_i_(MT_2_).

c NSB – no specific binding for up to 30 µM of tested compounds in the binding buffer.

The pIC_50_ and %Resp were mean ± SEM of 3–5 trials done in duplicates. The corresponding K_i_ values were calculated using the mean pIC_50_ values.

**Figure 3:**
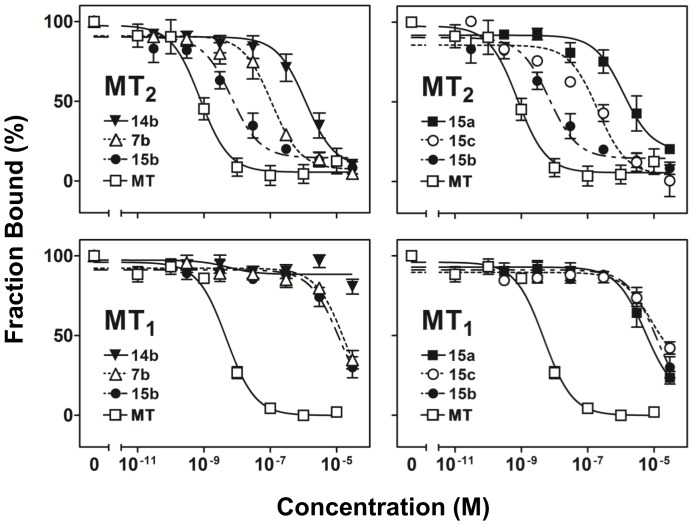
Competitive receptor binding curves of selective isoquinolinone derivatives. Intact CHO cells expressing MT_1_ or MT_2_ were incubated with [^3^H]melatonin with or without different concentrations of selected tested compounds or unlabeled melatonin. Data represented mean ± SEM of at least 3 different trials performed in duplicates, and normalized to the maximal binding values (in the absence of tested compound). Estimation of maximal displacement and IC_50_ and K_i_ were shown in Table 1.

The next group of compounds **14a–d** is 6-methoxy-isoquinolinones with a benzyloxyl or substituted benzyloxyl substituent at C7 position. They appeared to bind MT_2_ much weaker than the last group of substituted 7-methoxy-isoquinolinones **7a**–**7g**. The compound **14b** containing the 3-methoxybenzyloxyl substituent as of **7b** also exhibited the highest affinity toward MT_2_ receptor subtype among this group, but its affinity was significantly lower than **7b**. **14b** also completely displaced [^3^H]melatonin under the same experimental condition. The last group **15a–d** is 6-methoxy-isoquinolinones with a different substituent at C5 position. Similar to the previous two groups, **15b** bearing a 3-methoxybenzyloxyl substituent conferred the highest binding affinity toward MT_2_ among this group. **15b** displayed high MT_2_ affinity (K_i_ = 1.7 nM), only approximately one order lower than melatonin as determined in the same condition, but 1800-fold selectivity over MT_1_. Among the compounds bearing a 3,5-dimethoxybenzyloxyl group (**7c**, **14c** and **15c**), **15c** showed significantly higher affinity toward MT_2_ and almost complete displacement of [^3^H]melatonin. It also displayed 51-fold of selectivity toward MT_2_ than MT_1_. The order of compounds in terms of their MT_2_ binding affinity and selectivity in descending order was **15b**>**15c**>**15a** (Figure 3). Overall, the results of competitive binding assays showed that the presence of a 3-methoxybenzyloxyl group increased the affinity toward MT_2_ but not MT_1_, and its attachment position on the isoquinolinone scaffold was important for the overall binding affinity, with **15b** bearing a 3-methoxybenzyloxyl group at C5 being the most potent compound (Figure 3).

### Functional characterization of isoquinolinone-based melatoninergic ligands

**Figure 4:**
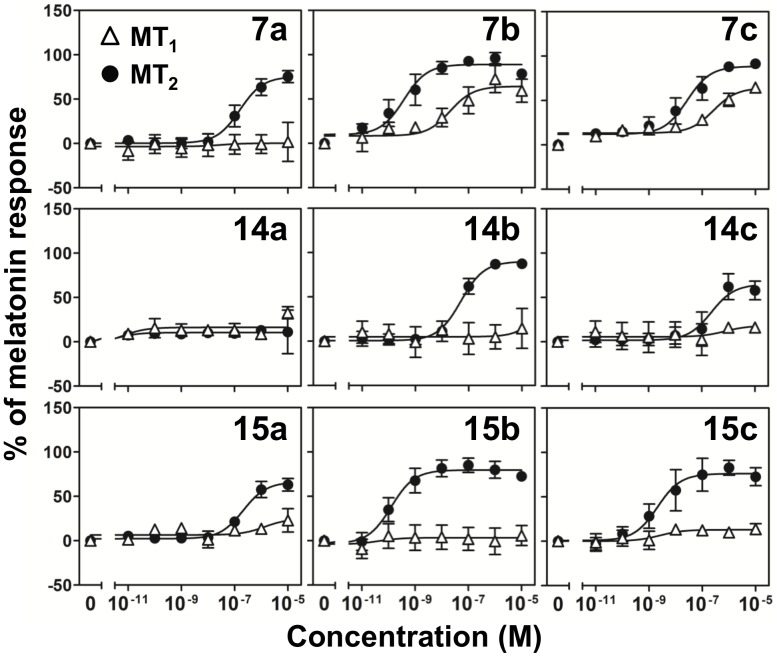
Stimulation of intracellular Ca^2+^ mobilization in CHO cells expressing MT_1_ or MT_2_ by isoquinolinone derivatives. CHO cells expressing MT_1_ or MT_2_ were subjected to the treatment of increasing doses of selected tested compounds. Data were mean of peak fluorescence signals ± SEM of at least 3 different trials performed in triplicates, and normalized to the maximal response elicited by melatonin (as 100%) and the minimal response of vehicle-treated cells (as 0%). Estimation of maximal responses and EC_50_ were tabulated in Table 2.

Individual melatonin receptor subtypes were stably expressed in Chinese hamster ovary (CHO) cells together with a chimeric G protein α subunit 16z25; the 16z25 chimera channels receptor activation signals to the mobilization of intracellular Ca^2+^ for real-time detection in a FLIPR [24]–[26]. The estimated EC_50_ and percentage activation of the melatonin-induced responses as deduced from the concentration-dependent stimulation induced by the various isoquinolinone compounds are summarized in Table 2. In the positive controls, melatonin induced robust dose-dependent responses in both MT_1_ and MT_2_-expressing CHO cells (MT_1_-CHO and MT_2_-CHO) with very similar EC_50_ in the sub-nM range, and melatonin induced a slightly more potent response in MT_1_-expressing cells as expected. Compound **12** partially activated both MT_1_ and MT_2_ receptors at 10 µM with a maximal response of 36% and 53%, respectively (data not shown), but for most of the tested compounds, no significant response could be induced in MT_1_-CHO cells at the maximal concentration tested (10 µM; Table 2). Exceptions are **7b** and **7c** where concentration response curves could be constructed (Figure 4) with EC_50_ values of 31.1 and 266 nM, respectively. Both compounds have a meta-methoxybenzyloxyl substitutent at C6 position of the isoquinolinone scaffold. Similar analogs with a meta-methoxybenzyloxyl group at either C7 (**14b** and **14c**) or C5 (**15b** and **15c**) position could not produce any activity at MT_1_. Again, **7b** outperformed **7c** to activate MT_1_ with an EC_50_ lower by an order of magnitude, which correlated with the results in the binding assay.

**Table 2:** Isoquinolinone-induced intracellular Ca^2+^ mobilization in MT_1_-CHO and MT_2_-CHO cells.

	MT_1_			MT_2_		
Compound	pEC_50_ ±SEM	EC_50_ (nM)	%Resp^a^ ±SEM	pEC_50_ ±SEM	EC_50_ (nM)	%Resp ±SEM
MT	9.85±0.21	0.16	100±26	9.23±0.07	0.6	100±30
**7a**	-	NSR^b^	-	6.79±0.30	163	84±20
**7b**	7.51±0.17	31.1	63±7	9.54±0.56	0.30	92±11
**7c**	6.58±0.29	266	65±10	7.68±0.59	21.0	90±16
**7d**	-	NSR	-	7.59±0.62	25.7	85±22
**7e**	-	NSR	-	-	NSR	-
**7f**	-	NSR	-	-	NSR	-
**7g**	-	NSR	-	6.96±0.41	110	83±6
**14a**	-	NSR	-	-	NSR	-
**14b**	-	NSR	-	7.31±0.19	48.9	96±16
**14c**	-	NSR	-	6.64±0.40	228	71±17
**14d**	-	NSR	-	-	NSR	-
**15a**	-	NSR	-	6.67±0.05	215	70±9
**15b**	-	NSR	-	9.84±0.28	0.14	89±20
**15c**	-	NSR	-	8.39±0.55	4.08	85±19
**15d**	-	NSR	-	6.89±0.48	128	40±17

a %Resp – Percentage responses of the melatonin (MT)-mediated stimulation of Ca^2+^ signal.

b NSR – no significant response for up to 30 µM of tested compounds in the binding buffer.

The pEC_50_ and %Resp were mean ± SEM of 3–5 trials done in duplicates. The corresponding EC_50_ values were calculated using the mean pEC_50_ values.

When assayed in MT_2_-CHO cells, most of the tested compounds elicited concentration-dependent stimulation of Ca^2+^ signals, and allowed for a detailed interpretation of their structure-function relationship. In the binding assay, among compounds **7a–7g** with a benzyloxyl or substituted benzyloxyl group at C6, **7b** showed the most outstanding binding to MT_2_. Expectedly, **7b** induced robust dose-dependent Ca^2+^ signals with an EC_50_ of 0.3 nM and its maximal response was very close to that of melatonin (Table 2). Although the apparent K_i_ values of the other C6-benzyloxyl substituted compounds were very close to each other (Table 1), they behaved differently in triggering Ca^2+^ signals. Compound **7c** with an additional methoxy group on the benzyloxyl branch was ∼70-fold less potent than **7b**. Further reduction in potency was observed for **7a** which was devoid of methoxy group on the benzyloxyl branch. Concentration response curves of **7a**–**7c** in both MT_1_ and MT_2_-expressing cells are shown in Figure 4. It is obvious that **7a** could trigger Ca^2+^ signals exclusively in MT_2_-CHO cells at around 1 µM, whereas the concentration of 7**c** to induce exclusive MT_2_ activation was even lower (<10 nM).

Very different responses were observed for another two mono-methoxybenzyloxyl derivatives **7d** and **7e**. By simply moving the methoxy group on the benzyloxyl branch from *meta* to *ortho* position, the ability of **7d** to trigger Ca^2+^ signal was reduced by 2 orders of magnitude (Table 2), indicating a stringent structural requirement for the MT_2_ selectivity and the importance of this particular methoxy group. The position effect was further manifested by the null response of **7e** – the *para*-methoxybenzyloxyl derivative. **7f** and **7g** are methylbenzyloxyl derivatives, analogous to **7e** and **7c** for the number and position of the methyl substituent. In terms of EC_50_ and maximal response of Ca^2+^ signals, **7f** and **7g** behaved very similarly to **7e** and **7c**, respectively.

Compounds **14a–d** with a benzyloxyl or substituted benzyloxyl group at C7 had the weakest binding affinities (Table 1), and so were their abilities to trigger Ca^2+^ signals. The best of this group, **14b** with a 3-methoxybezyloxyl substituent similar to **7b** showed an EC_50_>160- fold of **7b**. Compound **14c** was 11-fold less potent than **7c**, and **14a** and **14d** were basically inactive. A comparison of the concentration-dependent responses shown in Figure 4 clearly showed the much weaker responses of C7-benzyloxyl substituted compounds. Although both **14b** and **14c** bound and activated MT_2_ receptor, **14c** displayed only partial efficacy in FLIPR assay.

Compounds **15a–d** represented the best group of isoquinolinones as they were all capable of activating MT_2_ exclusively with high potency. The 3,5-dimethoxybenzyloxyl derivative **15c** was 5-fold more potent than **7c**, whereas the potency of **15a** was essentially indistinguishable from **7a**. The 3-methoxybenzyloxyl derivative **15b** possessed an EC_50_ comparable to that of melatonin toward MT_2_ and was totally inactive at MT_1_ (Table 2), further indicating that the importance of the presence of a single methoxy group on the *meta* position of the benzyloxyl branch in subtype selectivity. Its subtype selectivity was obviously better than **7b** (Figure 4, middle column). Interestingly, **15d** was the only 4-methylbenzyloxyl derivative (c.f. **7f** and **14d**) able to induce a receptor-mediated Ca^2+^ signal concentration-dependently, suggesting that MT_2_ receptor has a greater tolerance for substituent extending off the C5 position of the isoquinolinone scaffold.

**Figure 5:**
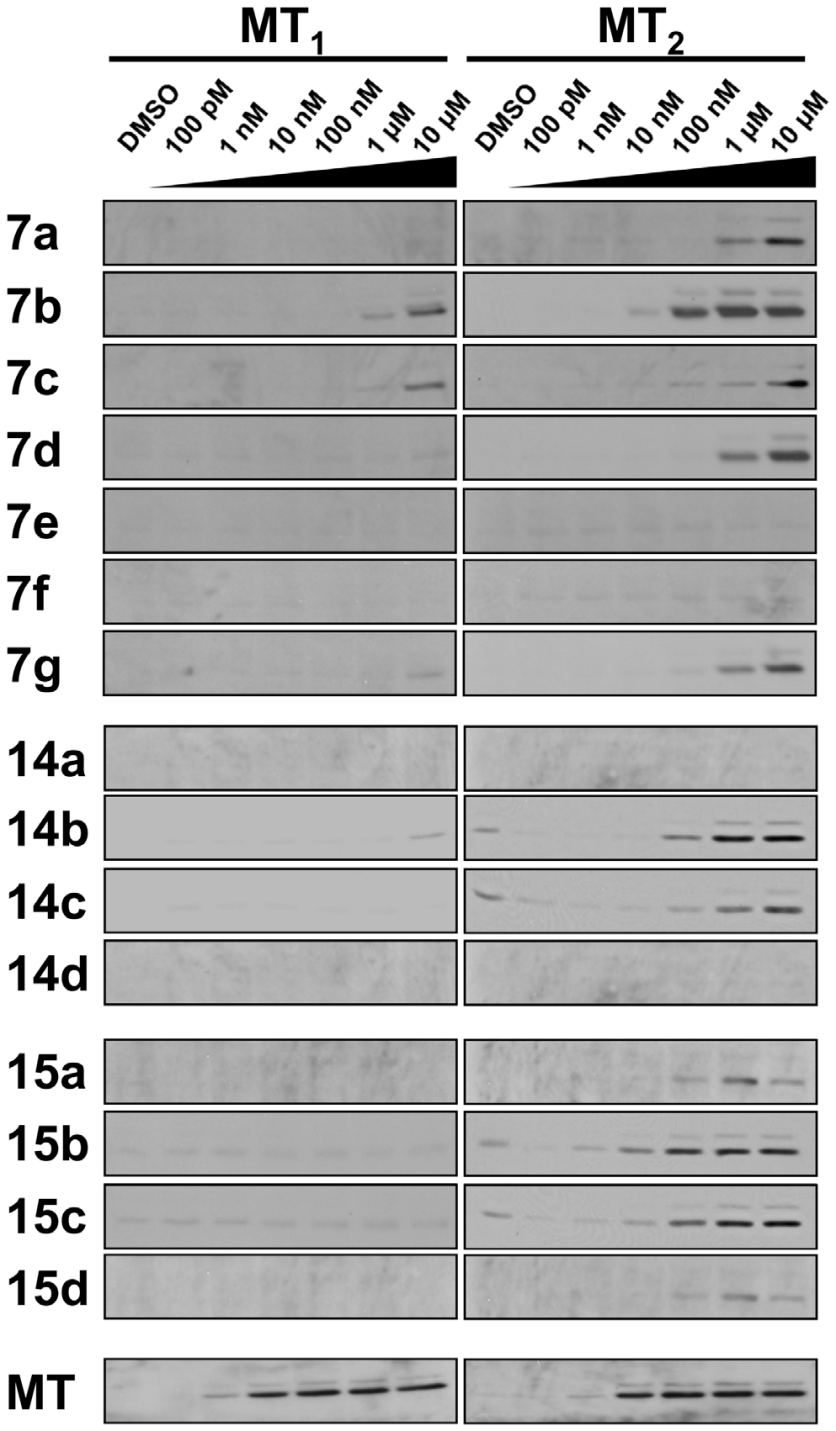
Phosphorylation of ERK induced by isoquinolinone derivatives. CHO cells expressing MT_1_ or MT_2_ were serum-starved before treating with the indicated concentrations of melatonin or individual tested compounds. Resolved proteins were electrotransferred for immunodetection using phosphorylated ERK-specific antibody. Total amount of ERK was also detected similarly and no observable change of their expression levels has been found for all the treatments (not shown). Three individual trails yielded similar results as the representative blots shown in the figure.

Melatonin can induce phosphorylation of extracellular signal-regulated protein kinases (ERK) in both MT_1_-CHO and MT_2_-CHO cells due to the presence of the 16z25 chimera. As shown in Figure 5, ERK phosphorylation became detectable when >1 nM of melatonin was added to either cell lines, indicating similar potencies of melatonin for both receptor subtypes. The total amount of ERK in the cell lysates loaded into the gels was monitored using a specific antibody and none of the treatment had any effect on the total amount of ERK (data not shown). Resembling the results in FLIPR assays, only **7b** and **7c** could induce weak ERK phosphorylation in MT_1_-CHO cells at a concentration of >1 µM, and all the other tested compounds were ineffective. In contrast, most of the tested compounds activated ERK phosphorylation in MT_2_-CHO cells in a concentration-dependent manner but with different potencies. The rankings of each group of tested compounds with the same position substituted with different modified benzyloxyl groups were generally very similar to those obtained in the FLIPR assay. Compounds bearing a 3-methoxybenzyloxyl substituent (**7b**, **14b**, and **15b**) were the most effective ones in each group, followed by compounds bearing a 3,5-dimethoxybenzyloxyl substituent (**7c**, **14c**, and **15c**). While the difference between 3-methoxybenzyloxyl (**7b**, **14b**, and **15b**) and 3,5-di-methoxybenzyloxyl (**7c**, **14c**, **15c**) derivatives were obvious, the difference of the ERK phosphorylation responses induced by **15b** was only slightly better than **15c**. These results further suggested that the 3,5-dimethoxybenzyloxyl substituent was better tolerated when located at C5 of the isoquinolinone scaffold. Compounds bearing a benzyloxyl (**7a** and **15a**) or a 4-methylbenzyloxyl substituent (**14d** and **15d**) at C6 or C5 could only stimulate ERK phosphorylation very weakly, and compounds (**14a** and **14d**) bearing the same substituents at C7 were essentially inactive.

**Figure 6:**
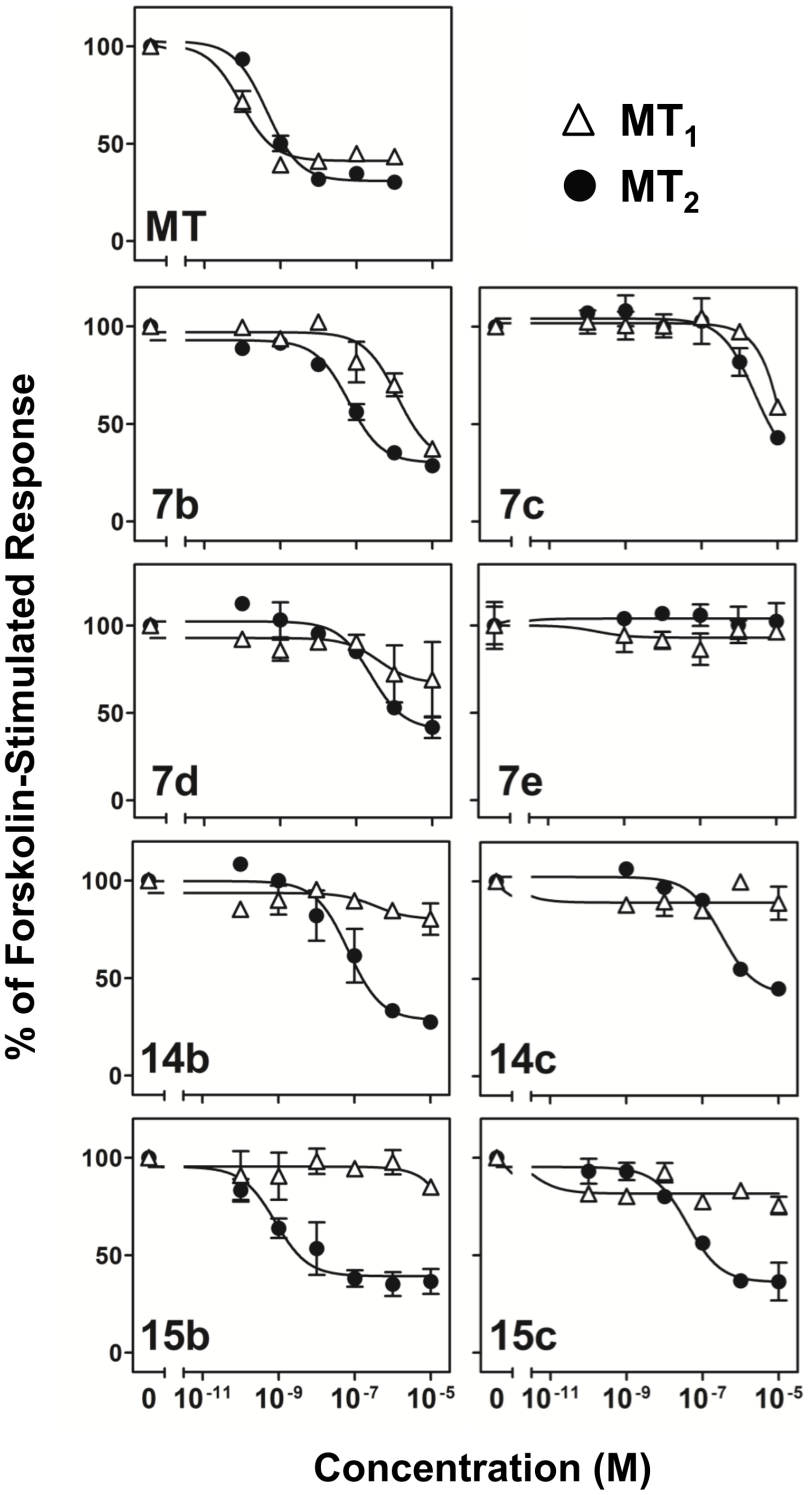
Isoquinolinone derivative-induced inhibition of forskolin-stimulated cAMP production. CHO cells expressing MT_1_ or MT_2_ were treated with 50 µM forskolin and increasing concentrations of individual tested compounds as indicated at the lower left corner of each plot. All the responses were expressed as the percentage of that induced by forskolin alone (as 100%). Estimation of maximal inhibition and IC_50_ were tabulated in Table 3.

Both MT_1_ and MT_2_ are classified as G_i_-coupled receptors with their activation leading to inhibition of intracellular cAMP production. The ability of the tested compounds to activate the endogenously expressed G_i_-mediated inhibition of cAMP production was therefore examined. Melatonin induced 60–70% inhibition of the cAMP level elevated by forskolin, a direct activator of adenylyl cyclase, in both MT_1_-CHO and MT_2_-CHO cells with sub-nM IC_50_'s (Table 3), reflecting intact G_i_-dependent regulatory pathways in both cell lines. Eight selected isoquinolinones were examined for their dose-dependent inhibition of cAMP accumulation (Figure 6). In MT_1_-CHO cells, only **7b** and **7d** showed observable inhibition of cAMP levels with estimated IC_50_ values in the single digit or sub-µM range. Other tested compounds were basically unable to cause any inhibition. Similar to the previous assays in MT_2_-CHO cells, derivatives bearing a 3-methoxybenzyloxyl substituent (**7b**, **14b** and **15b**) outperformed other subgroup members bearing another substituent at the same position of the isoquinolinone scaffold. Both **7b** and **14b** showed similar IC_50_'s and their maximal percentage inhibition resembled that of melatonin, but **14b** showed a better selectivity toward MT_2_. Compound **15b** has an IC_50_ close to that of melatonin at MT_2_ but was completely inactive at MT_1_ (Table 3). The derivatives containing a 3,5-dimethoxybenzyloxyl substituent showed a progressive decrease of IC_50_ in the order of **7c**, **14c** and **15c**, further manifesting the positional effect of the benzyloxyl substituent in which C5 appeared to be the most desirable position for a highly MT_2_-selective compound. Overall, the results in all three different functional assays indicated that a 3-methoxybenzyloxyl substitutent at C5 of the isoquinolinone scaffold yielded a novel MT_2_-selective melatoninergic agonist with single digit or sub-nM activities.

**Table 3:** Isoquinolinone-induced inhibition of cAMP production in MT_1_-CHO and MT_2_-CHO cells.

	MT_1_			MT_2_		
Compound	pIC_50_ ±SEM	IC_50_ (nM)	%Inh_max_ a ±SEM	pIC_50_ ±SEM	IC_50_ (nM)	%Inh_max_ ±SEM
MT	10.05±0.13	0.090	59±2	9.36±0.09	0.44	69±2
**7b**	5.92±0.21	1201	69±8	7.20±0.12	92.9	70±3
**7c**	-	NSR^b^	-	5.61±0.24	2460	72±3^c^
**7d**	6.40±1.11	396	33±13	6.61±0.18	247	59±5
**14b**	-	NSR	-	7.16±0.24	69.4	71±6
**14c**	-	NSR	-	6.49±0.11	326	58±3
**15b**	-	NSR	-	9.09±0.25	0.81	61±4
**15c**	-	NSR	-	7.39±0.16	40.6	64±3

The pIC_50_ and %Inh_max_ were mean ± SEM of 2 trials done in duplicates. The corresponding IC_50_ values were calculated using the mean pIC_50_ values.

a %Inh_max_ – Maximal percentage inhibition of the forskolin-elevated cAMP level.

b NSR – no significant response for up to 10 µM of tested compounds in the assay buffer.

c Extrapolated %Inh_max_ from non-linear regression.

### Identification of potential MT2-selective antagonists

**Figure 7:**
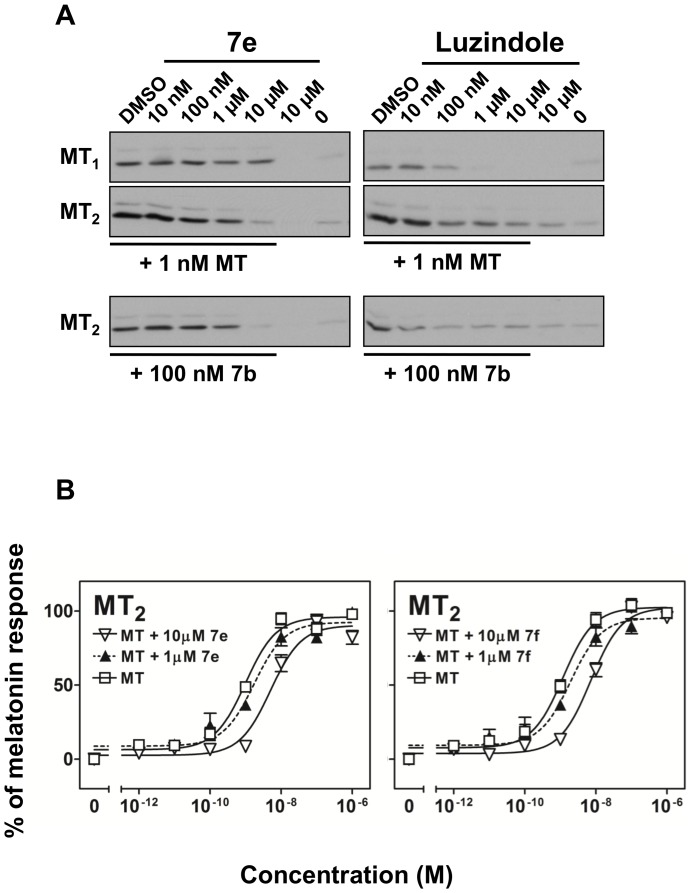
Blockade of ERK phosphorylation and Ca^2+^ mobilization by an isoquinolinone-based melatoninergic antagonist. (A) CHO cells expressing MT_1_ or MT_2_ were treated with the indicated concentrations of **7e** or luzindole in the absence or presence of a fixed concentration of melatonin (MLT) (for both MT_1_ or MT_2_) or **7b** (for MT_2_ only). Other experimental details were as to the legend of Figure 5. Data shown were representative blots of three separate trials. (B) CHO cells expressing MT_2_ were treated with increasing concentrations (1 ρM – 1 µM) of melatonin in the absence or presence of 10 µM or 1 µM of **7e** or **7f**. Other experimental details were as to the legend of Figure 4.

Among the low affinity tested compounds (**7a**, **7d**, **7e**, **7f** and **7g**), **7e** and **7f** did not show any activities in all three functional assays (Tables 2, 3, Figures 5, 6, and S2). This observation prompted us to explore if these *para*-substituted benzyloxyl derivatives possess antagonistic activity towards MT_2_. In the presence of 1 nM of melatonin (MLT), increasing doses of **7e** suppressed the agonist-induced ERK phosphorylation in MT_2_-CHO but not in MT_1_-CHO cells (Figure 7A, left hand side, upper two blots). The level of ERK phosphorylation was reduced to almost the basal level when 10 µM of **7e** was present (lane 5 versus 7). Moreover, ERK phosphorylation induced by the application of a structurally closely related agonist **7b** (100 nM) was also significantly suppressed by **7e** in MT_2_-CHO cells (at 10 µM; Figure 7A, lower left hand panel). Despite having a similar binding affinity as **7e**, *para*-methylbenzyloxyl derivative **7f** demonstrated a weaker antagonistic activity, with partial inhibition of the MT_2_ response at 10 µM (Figure S3). To confirm competitive antagonism, MT_2_-CHO cells were treated with melatonin in the absence or presence of **7e** or **7f**. A parallel right shift of the melatonin dose-response curve was observed in FLIPR assay upon preincubation of **7e** or **7f** (Figure 7B), displaying a competitive interaction between melatonin and the isoquinolinone antagonists. A typical MT_2_-selective antagonist luzindole was employed in the control experiment (Figure 7A, right hand side). Luzindole blocked the ERK phosphorylation induced by either MLT in both MT_1_-CHO and MT_2_-CHO cells or **7b** in MT_2_-CHO cells in a more effective manner, which was consistent to the higher affinity of luzindole towards both MT_1_ (K_i_ = 102 nM) and MT_2_ (K_i_ = 2.9 nM) under our experimental conditions. In MT_1_-CHO cells, luzindole completely eliminated basal ERK phosphorylation at 1 µM. However, the observed basal ERK phosphorylation was constantly enhanced in MT_2_-CHO cells treated with luzindole alone. In fact, a weak but significant MT_2_-selective agonistic activity of luzindole was also detected in FLIPR assay (Figure S2). In conclusion, the results suggested that **7e** and **7f** represent novel competitive MT_2_-selective antagonists with relatively low affinity.

## Discussion

The results of the present study demonstrate that substituted isoquinolinones possess melatoninergic activities with selectivity toward MT_2_. A substituted benzyloxyl group on three carbons (C5>C6>C7 in descending order of binding affinity) of the isoquinolinone scaffold rendered differential affinity toward MT_2_, whereas most of the derivatives were basically unable to bind MT_1_. Compound **15b** with a 3-methoxybenzyloxyl group at C5 position was the most potent MT_2_-selective agonist with a binding affinity of 1.66 nM toward MT_2_ and a selectivity of 1800-fold over MT_1_. Benzyloxyl group bearing one meta-methoxy outperformed two meta-methoxy substituents, suggesting the MT_2_ ligand binding pocket was very sensitive to steric hindrance. This was further demonstrated by the distinctive behaviors of **7d** and **7e**: while *ortho*-methoxy derivative **7d** was tolerated in maintaining the agonist property, *para*-methoxybenzyloxyl group attached to C6 position generated an MT_2_-selective weak antagonist **7e**.

The extended ethylamido chain is a key feature for most of the previously known melatoninergic compounds [27], [28]. Farce et al. suggested that both the carbonyl oxygen and the amine proton of the amide are engaged in hydrogen bonds with the serine residues in the putative binding site, thus governing the compounds' binding affinity toward both melatonin receptors [29]. The lack of such extended ethylamido chain in our tested compounds might cause a significant reduction of binding affinity when compared with melatonin, but at the same time the importance of other structural features corresponding to receptor binding affinity could become more apparent.

Our lead compound **12**, which does not contain an alkyladimoethyl chain or an additional aromatic group, showed very weak activities toward both MT_1_ and MT_2_ receptors as shown in Ca^2+^ mobilization assay. By attaching an aromatic 3-methoxyphenyl ring through CH_2_-O chain to isoquinolinone at C7, compound **14b** partially compensated the effect caused by the lack of an alkyladimoethyl chain and exhibited fair activity toward MT_2_. When the aromatic 3-methyoxylphenyl was switched from C7 to C6 or C5 position, the activities (compound **7a** and **15b**) towards MT_2_ were further enhanced. These results demonstrated the importance of the aromatic 3-methoxyphenyl group and its relative orientations on the isoquinolinone scaffold. When the aromatic 3-methoxyphenyl group is attached at C5 position of isoquinolinone through CH_2_-O chain, it (compound **15b**) provided the most potent activity and highest selectivity towards MT_2_. Melatonin ligands bearing additional aromatic rings and no alkylamidoethyl chain have been identified previously. Karageorge et al developed a series of tetrahydroisoquinoline derivatives as melatonin MT_2_ receptor antagonists after following a lead compound identified by high throughput screening [30]. These compounds and our isoquinolinone derivatives may bind to melatonin MT_2_ at a similar site through interacting with the additional aromatic group.

The flexibility of the ethylamido chain allows diverse conformational arrangements in order to fit the ligand into the binding pockets of the two melatonin receptors in a slightly different manner. In previous mutagenesis studies, MT_1_ and MT_2_ receptors displayed distinct tolerance to conserved serine mutations, which hinted at structural divergence between the two subtypes. [31], [32]. It has been shown that a substituent at C2 position of melatonin (either halogen atom or phenyl group, which is either lying on the same plane of the indole ring or has only very limited degree of freedom) can enhance the binding affinity for both receptor subtypes [33]. However, a 2-benzyl substituent which lies away from the plane of the indole scaffold of melatonin specifically enhances MT_2_ binding only [34]. It means that an additional binding cavity, which is away from the plane of the indole ring and is able to accommodate the aromatic substituent, may be present in MT_2_ but not MT_1_. Such a cavity is most likely formed by a large group of hydrophobic amino acid side chains located at TM5 (Y200, V204, V205, H208), TM6 (W264, L267, Ile270) and TM7 (F290, Y294, Y298) [35]. This might explain why our isoquinolinone compounds with a 3-methoxybenzyloxyl substituent show modest affinity towards MT_2_ even in the absence of the ethylamido chain.

With the 2-benzyl moiety of luzindole resembling the benzyloxyl group of **7a**, **14a** and **15a**, the partial agonist activity of luzindole, as illustrated in Ca^2+^ mobilization (Figure S2) and ERK phosphorylation assays (Figure 7A), further support the notion that additional aromatic ring may be important for MT_2_ binding. Luzindole has previously been suggested as a MT_2_ partial agonist *in vivo* based on its melatonin-like effect to depress the excitatory postsynaptic potentials evoked by mouse hippocampal neurons [36] and the firing of rat medial vestibular nucleus neurons [37]. The coexistence of an aromatic group and the alkylamidoethyl chain at juxtaposition on either a benzofuran [38] or simply phenyl scaffold [26] gives rise to very potent MT_2_-selective ligands, suggesting that the two key features bind to non-overlapping regions of MT_2_ and produce synergistic effect on receptor binding.

## Conclusions

This study has provided new insights on the design of subtype selective melatonin receptor agonists and antagonists. Isoquinolinone derivatives are a novel category of melatoninergic ligands without an extended ethylamido side chain. Both agonists and antagonists selective to MT_2_ have been identified, and the results have refined our understanding on the importance of the aromatic substituent on the receptor subtype selectivity and functional characteristics of melatoninergic compounds. A 3-methoxybenzyloxyl substituent at C5 or C6 position of isoquinolinone scaffold rendered MT_2_ selectivity. The number and position of methoxy group on the benzyl ring dictates the binding affinity as well as functionality of the ligands. Further investigation on the stability and fine-tuning of the structure-activity relationship of isoquinolinone-based compounds may therefore represent a promising design strategy for melatonin receptor subtype-specific therapeutic agents.

## Experimental section

### Synthesis

All reagents and solvents were purchased from commercial sources and used as received unless specified. Dry methylene chloride was distilled from CaH_2_. Flash chromatography was performed with Merck silica gel 60 (230–400 mesh), and reaction progress was determined by thin-layer chromatography (TLC) on silica gel plates. Yields were based on purified compounds and were not optimized. Melting points were determined on Aldrich Mel-TEMP II capillary melting-point apparatus and uncorrected. NMR spectra were recorded on a Joel EX 400, or a Varian Mercury 300 spectrometer. Chemical shifts of the ^1^H-NMR were referenced to residual solvent (chloroform at 7.26 ppm) or TMS (0.00 ppm). Chemical shifts of ^13^C-NMR were referenced to CDCl_3_ at 77.00 ppm. ESI-MS was taken on a Thermo-Finnigan LCQ Classic ion trap mass spectrometer; only molecular ions (M+1) were given. HR-MS recorded on MALDI Micro MX Mass Spectrometer by Waters MICROMASS. Purity and characterization of compounds were established by both NMR and MS. The purity of the final compounds was >95% by NMR and HPLC analysis.

#### (2-Benzenesulfinylethyl)-methylamine (2)

Phenylvinyl sulfoxide (**1**, 3 ml, 22.4 mmol) was added to a solution of methylamine in THF (2.0 M, 16.8 ml, 33.7 mmol). The mixture was sealed and stirred at room temperature for 18 hours. The solvent was removed under vacuum after the reaction, and the crude product was purified by flash column chromatography on silica gel (MeOH∶CH_2_Cl_2_∶NH_3_ = 10∶90∶1) to yield the desired product **2** (2.9 g, 17.1 mmol, 76%), which was used directly without further purifications.

#### N-(2-Benzenesulfinylethyl)-4-hydroxy-3-methoxy-N-methylbenzamide (4)

Diisopropyl carbodiimide (2.6 ml, 20.6 mmol) was added dropwise to a solution of compound **2** (2.9 g, 17.1 mmol), 4-hydroxy-3-methoxybenzoic acid (**3**, 3.5 g, 20.6 mmol) and hydroxybenzotriazole (2.8 g, 20.6 mmol) dissolved in 80 ml CH_2_Cl_2_ under N_2_. The reaction was stirred at room temperature for 12 hours, and the white urea solid was removed by filtration. The filtrate was concentrated and the crude product was purified by flash column chromatography on silica gel (MeOH/CH_2_Cl_2_ = 1∶25) yielding **4** (4.8 g, 14.4 mmol, 84%) as a white solid. ^1^H-NMR (400 MHz, CDCl_3_) δ 7.64 (1H, dd, *J* = 8.4, 1.2 Hz), 7.56 (1H, d, *J* = 1.2 Hz), 7.30 (5H, m), 6.93 (1H, d, *J* = 8.4 Hz), 4.49 (2H, t, *J* = 5.6 Hz), 3.92 (3H, s), 3.31 (2H, t, *J* = 5.6 Hz), 2.94 (3H, s).

#### Acetic acid 4-[(2-acetoxy-2-phenylsulfanylethyl)-methylcarbamoyl]-2-methoxyphenyl ester (5)

Compound **4** (4.8 g, 14.4 mmol) was dissolved in 40 ml acetic anhydride and refluxed for 6 hours. The excessive acetic anhydride was then removed under reduced pressure. The resulting residue was treated with saturated NaHCO_3_, extracted with CH_2_Cl_2_, dried and concentrated. The crude product was purified by flash column chromatography on silica gel (ethyl acetate∶hexane = 1∶1) yielding **5** (6.1 g, 100%) as a brown oil, which was used in the next step without further purification. ^1^H-NMR (400 MHz, CDCl_3_): δ 7.53 (1H, m), 7.32 (4H, m), 7.01 (2H, m), 6.90 (1H, m), 6.50 (1H, s), 3.88 (2H, m), 3.81 (3H, s), 3.03 (3H, s), 2.32 (3H, s), 2.09 (3H, s).

#### 6-Hydroxy-7-methoxy-2-methylisoquinolin-1(2H)-one (6)

*p*-Toluenesulfonic acid monohydrate (12.2 g, 64.1 mmol) was added to a solution of compound **5** (crude 6.1 g) dissolved in 70 ml toluene. The mixture was refluxed under N_2_ for 40 min, and the solvent was then removed under reduced pressure. The resulting residue was neutralized with saturated NaHCO_3_, and extracted with CH_2_Cl_2_ for several times until TLC of the aqueous phase did not show the desired product. The combined CH_2_Cl_2_ extract was dried and concentrated. The crude product was purified by flash column chromatography on silica gel (MeOH∶CH_2_Cl_2_ = 1∶25) yielding **6** (2.1 g, 10.2 mmol, 71%) as a white solid. ^1^H-NMR (400 MHz, CDCl_3_): δ 7.82 (1H, s), 6.99 (1H, s), 6.98 (1H, d, *J* = 7.2 Hz), 6.38 (1H, d, *J* = 7.2 Hz), 3.99 (3H, s), 3.60 (3H, s). ^13^C-NMR (100 MHz, CDCl_3_): δ 162.0, 150.7, 147.8, 132.9, 130.4, 118.9, 109.4, 106.9, 106.0, 55.7, 36.9.

#### N-(2-Benzenesulfinyl-ethyl)-3-hydroxy-4-methoxy-N-methylbenzamide (9)

Diisopropyl carbodiimide (7.9 ml, 50.3 mmol) was added dropwise to a solution of compound **2** (8.38 g, 45.7 mmol), compound **8** (8.46 g, 50.3 mmol) and hydroxybenzotriazole (6.84 g, 50.3 mmol) in a mixture of CH_2_Cl_2_ (150 ml) and DMF (40 ml) under N_2_. After stirring at room temperature for 2 days, the reaction was stopped and concentrated under reduced pressure. CH_2_Cl_2_ was added to the residue and the white urea salt was filtered. The filtrate was treated with saturated NH_4_Cl, extracted with CH_2_Cl_2_, dried and concentrated. The crude product was purified by flash column chromatography on silica gel (MeOH∶CH_2_Cl_2_ = 1∶20) yielding **9** (18.83 g, 84.3%) as a foam solid, which was used in the next step without further purification.

#### N-(2-Benzenesulfinyl-ethyl)-3-benzyloxy-4-methoxy-N-methylbenzamide (10)

Benzyl bromide (8.1 ml, 68.6 mmol) and K_2_CO_3_ (9.5 g, 68.6 mmol) were added to the crude product **9** (18.83 g, 56.5 mmol) in 100 ml DMF. The mixture was stirred at room temperature for 17 hours. The solvent was removed under reduced pressure. Water was added to the residue and then extracted with ethyl acetate for 3 times. The combined ethyl acetate extract was dried, filtered and concentrated. The crude product was purified by flash column chromatography with silica gel (ethyl acetate∶hexane, from 3∶2 to 4∶1, then ethyl acetate) yielding **10** (17.7 g, 41.8 mmol, 91% for two steps) as a pale yellow oil.

#### General procedure for the preparation of compounds 12 and 13

2,4,6-Collidine (16.6 ml, 125.4 mmol) was added to a solution of the product **10** (17.7 g, 41.8 mmol) in 210 ml CH_2_Cl_2_ under N_2_ at 0°C, followed by adding TFAA (29.5 ml, 208.95 mmol) dropwise. After stirring for 30 min, the reaction was quenched by slow addition of 180 ml 10% K_2_CO_3_. The mixture was then warmed to room temperature. The layers were separated and the aqueous layer was extracted twice with CH_2_Cl_2_. The combined CH_2_Cl_2_ was washed twice with 10% HCl, dried, filtered, concentrated and then dissolved in 210 ml toluene. *p*-Toluenesulfonic acid monohydrate (39.75 g, 209.0 mmol) was then added and the mixture was refluxed for 40 min. The reaction was cooled to room temperature, and saturated NaHCO_3_ was added until pH was 8. The aqueous layer was separated and extracted with CH_2_Cl_2_ several times until TLC of the aqueous layer did not show the desired products. The combined organic layers were dried, filtered and concentrated. The crude products were purified by flash column chromatography on silica gel (ethyl acetate∶hexane∶ammonia = 60∶40∶1) yielding **13** and (ethyl acetate∶hexane = 4∶1) **12** as white solids.

#### 7-Hydroxy-6-methoxy-2-methylisoquinolin-1(2H)-one (12)

The crude products were purified by flash column chromatography on silica gel (ethyl acetate∶hexane = 4∶1) yielding **12** *(3.2 g, 15.6 mmol, 37% from* ***10*** *)* as a white solid. ^1^H-NMR (400 MHz, CDCl_3_) δ 7.78 (1H, s), 6.98 (1H, d, *J* = 7.4 Hz), 6.87 (1H, s), 6.46 (1H, d, *J* = 7.4 Hz), 3.98 (3H, s), 3.60 (3H, s).

#### 5-Hydroxy-6-methoxy-2-methylisoquinolin-1(2H)-one (13)

The crude products were purified by flash column chromatography on silica gel (ethyl acetate∶hexane∶ammonia = 60∶40∶1) yielding **13** *(2.5 g, 10 mmol, 24% from* ***10*** *)* as a white solid. ^1^H-NMR (400 MHz, CDCl_3_) δ 7.96 (1H, d, *J* = 8.8 Hz), 7.03 (1H, d, *J* = 8.8 Hz), 6.97 (1H, d, *J* = 7.6 Hz), 6.83 (1H, d, *J* = 7.6 Hz), 3.86 (3H, s), 3.55 (3H, s).

#### Common alkylation procedure for the preparation of compounds 7a–g, 14a–d and15a–d

Desired alkyl bromide (∼1.3 equiv.) and K_2_CO_3_ (2 equiv.) was added to a solution of 1 equiv. of either **6**, **12** or **13** in DMF (0.2 M), and the mixture was stirred overnight at room temperature. Water was added after the reaction was completed as shown by TLC, and the mixture was extracted with ethyl acetate, dried, concentrated and finally purified by column chromatography to give the desired products.

#### 6-Benzyloxy-7-methoxy-2-methylisoquinolin-1(2H)-one (7a)

The crude product was purified by flash column chromatography on silica gel (ethyl acetate∶petroleum ether = 17∶3) yielding **7a** (98%) as a white solid. mp: 102–104°C. ESI-MS: 296.23 (M+1). HR-TOF-MS: calculated for C_18_H_17_NO_3_, 296.1287 (M+1); found 296.1296 (M+1). ^1^H-NMR (300 MHz, CDCl_3_): δ 7.82 (1H, s), 7.45–7.48 (2H, m), 7.32–7.41(3H, m), 6.96 (1H, d, *J* = 7.4 Hz), 6.88 (1H, s), 6.34 (1H, d, *J* = 7.4 Hz), 5.23 (2H, s), 4.00 (3H, s), 3.58 (3H, s). ^13^C-NMR (75 MHz, CDCl_3_): δ161.7, 152.3, 149.6, 136.1, 132.3, 130.9, 128.6, 128.1, 127.2, 107.7, 105.5, 70.7, 56.1, 37.0.

#### 7-Methoxy-6-(3-methoxy-benzyloxy)-2-methylisoquinolin-1(2H)-one (7b)

The crude product was purified by flash column chromatography on silica gel (ethyl acetate∶petroleum ether, from 3∶2 to 9∶1) yielding **7b** (100%) as a pale yellow solid. mp: 85–87°C. ESI-MS: 326.33 (M+1), 348.23 (M+Na). HR-TOF-MS: calculated for C_19_H_19_NO_4_, 326.1392 (M+1); found 326.1404 (M+1). ^1^H-NMR (300 MHz, CDCl_3_): δ 7.80 (1H, s), 7.28 (1H, t, *J* = 7.8 Hz), 7.01–7.04 (2H, m), 6.92 (1H, d, *J* = 6.3 Hz), 6.86–6.83 (1H, d, *J* = 8.8 Hz), 6.31 (1H, d, *J* = 7.2 Hz), 5.19 (2H, s), 3.98 (3H, s), 3.78 (3H, s), 3.55 (3H, s). ^13^C-NMR (100 MHz, CDCl_3_): δ 161.7, 159.7, 152.1, 149.4, 137.6, 132.2, 130.8, 129.6, 120.1, 119.2, 113.4, 112.6, 107.7, 107.6, 105.3, 70.4, 56.0, 55.0, 36.9.

#### 6-(3,5-Dimethoxy-benzyloxy)-7-methoxy-2-methylisoquinolin-1(2H)-one (7c)

ESI-MS: 356.27 (M+1), 378.13 (M+Na). HR-TOF-MS: calculated for C_20_H_21_NO_5_, 356.1498 (M+1); found 356.1514 (M+1). ^1^H-NMR (400 MHz, CDCl_3_): δ 7.82 (1H, s), 6.97 (1H, d, *J* = 7.2 Hz), 6.87 (1H, s), 6.61 (2H, s), 6.40 (1H, s), 6.34 (1H, d, *J* = 7.2 Hz), 5.19 (2H, s), 4.01 (3H, s), 3.79 (6H, s), 3.59 (3H, s). ^13^C-NMR (100 MHz, CDCl_3_): δ 160.9, 152.1, 149.5, 138.5, 132.2, 130.8, 120.3, 107.8, 107.7, 104.5, 104.9 (2), 99.8, 70.7, 56.2, 55.4 (2), 37.1.

#### 7-Methoxy-6-(2-methoxy-benzyloxy)-2-methylisoquinolin-1(2H)-one (7d)

The crude product was purified by flash column chromatography on silica gel (ethyl acetate∶hexane = 7∶3) yielding **7d** (100%) as a white solid. mp: 150–152°C. ESI-MS: 326.27 (M+1). HR-TOF-MS: calculated for C_19_H_19_NO_4_, 326.1392 (M+1); found 326.1398 (M+1). ^1^H-NMR (400 MHz, CDCl_3_): δ 7.82 (1H, s), 7.48 (1H, d, *J* = 6.4 Hz), 7.30 (1H, m), 6.91–7.00 (4H, m), 6.35 (1H, d, *J* = 7.1 Hz), 5.30 (2H, s), 4.01 (3H, s), 3.89 (3H, s), 3.59 (3H, s). ^13^C-NMR (100 MHz, CDCl_3_): δ 161.8, 156.5, 152.4, 149.6, 132.4, 130.8, 128.9, 128.2, 124.4, 120.7(2), 120.1, 110.2, 107.7, 105.6, 65.7, 56.2, 55.4, 37.1.

#### 7-Methoxy-6-(4-methoxy-benzyloxy)-2-methylisoquinolin-1(2H)-one (7e)

The crude product was purified by flash column chromatography on silica gel (ethyl acetate∶hexane, from 3∶2 to 4∶1) yielding **7e** (98%) as a white solid. mp: 170–172°C. ESI-MS: 326.27 (M+1). HR-TOF-MS: calculated for C_19_H_19_NO_4_, 326.1392 (M+1); found 326.1404 (M+1). ^1^H-NMR (300 MHz, CDCl_3_): δ 7.81 (1H, s), 7.39 (1H, d, *J* = 6.9 Hz), 6.89–6.96 (m, 4H), 6.34 (1H, d, *J* = 7.2 Hz), 5.15 (2H, s), 3.98 (3H, s), 3.80 (3H, s), 3.57 (3H, s). ^13^C-NMR (75 MHz, CDCl_3_): δ 161.8, 159.4, 152.3, 149.5, 132.3, 130.8, 129.0, 128.1, 120.1, 113.0, 107.7, 107.6, 105.4, 70.5, 56.0, 55.2, 37.0.

#### 7-Methoxy-2-methyl-6-(4-methyl-benzyloxy)-isoquinolin-1(2H)-one (7f)

The crude product was purified by flash column chromatography on silica gel (ethyl acetate∶petroleum ether = 4∶1) yielding **7f** (100%) as a white solid. mp: 120–122°C. ESI-MS: 310.27 (M+1), 332.20 (M+Na). HR-TOF-MS: calculated for C_19_H_19_NO_3_, 310.1443 (M+1); found 310.1448 (M+1). ^1^H-NMR (400 MHz, CDCl_3_): δ 7.81 (1H, s), 7.35 (2H, d, *J* = 7.6 Hz), 7.19 (2H, d, *J* = 7.6 Hz), 6.96 (1H, d, *J* = 7.2 Hz), 6.34 (1H, d, *J* = 7.2 Hz), 5.20 (2H, s), 3.99 (3H, s), 3.59 (3H, s), 2.35 (3H, s). ^13^C-NMR (100 MHz, CDCl_3_): δ 161.8, 152.3, 149.5, 137.8, 133.0, 132.3, 130.8, 129.3, 127.3, 120.1, 107.7(2), 105.5, 70.7, 56.2, 37.1, 21.3.

#### 6-(3,5-Dimethyl-benzyloxy)-7-methoxy-2-methylisoquinolin-1(2H)-one (7g)

ESI-MS: 324.26 (M+1). HR-TOF-MS: calculated for C_20_H_21_NO_3_, 324.1600 (M+1); found 310.1448 (M+1). ^1^H-NMR (400 MHz, CDCl_3_): δ 7.82 (1H, s), 7.07 (2H, s), 6.96 (2H, d, *J* = 7.2 Hz), 6.90 (1H, s), 6.35 (1H, d, *J* = 7.6 Hz), 5.16 (2H, s), 4.01 (3H, s), 3.59 (3H, s), 2.32 (6H, s). ^13^CNMR (75 MHz, CDCl_3_): δ 161.8, 152.4, 149.5, 138.2, 135.9, 132.3, 130.8, 129.7, 125.0 (2), 120.2, 107.7, 105.5, 70.9, 56.2, 37.1, 21.3.

#### 7-Benzyloxy-6-methoxy-2-methylisoquinolin-1(2H)-one (14a)

The crude product was purified by flash column chromatography on silica gel (ethyl acetate∶petroleum ether = 4∶1) yielding **14a** (100%) as a white solid. White solid. 100% yield. mp: 132–134°C. ESI-MS: 296.26 (M+1), 318.16 (M+Na). HR-TOF-MS: calculated for C_18_H_17_NO_3_, 296.1287 (M+1); found 296.1285 (M+1). ^1^H-NMR (300 MHz, CDCl_3_): δ 7.87 (1H, s), 7.49 (2H, m), 7.27–7.40 (3H, m), 6.95 (1H, d, *J* = 7.4 Hz), 6.85 (1H, s), 6.37 (1H, d, *J* = 7.3 Hz), 5.25 (2H, s), 3.95 (3 H, s), 3.56 (3 H, s). ^13^C-NMR (75 MHz, CDCl_3_): δ 161.8, 153.5, 148.2, 136.3, 132.6, 131.0, 128.5, 127.9, 127.5, 119.9, 109.2, 106.0, 105.3, 70.7, 55.9, 36.9.

#### 6-Methoxy-7-(3-methoxy-benzyloxy)-2-methylisoquinolin-1(2H)-one (14b)

The crude product was purified by flash column chromatography on silica gel (MeOH∶CH_2_Cl_2_ = 3∶200) yielding **14b** (78%) as a white solid. White solid. 78% yield. ESI-MS: 326.23 (M+1). HR-TOF-MS: calculated for C_19_H_19_NO_4_, 326.1392 (M+1); found 326.1391 (M+1). ^1^H-NMR (400 MHz, CDCl_3_): δ 7.86 (1H, s), 7.26–7.30 (1H, s), 7.05–7.08 (2H, m), 6.94 (1H, d, *J* = 7.2 Hz), 6.83–6.85 (2H, m), 6.36 (1H, d, *J* = 7.2 Hz), 3.94 (3H, s), 3.79 (3H, s), 3.54 (3H, s). ^13^C-NMR (75 MHz, CDCl_3_): δ 161.6, 159.6, 153.4, 148.1, 137.8, 132.6, 131.0, 129.5, 119.8, 119.7, 113.5, 112.8, 109.2, 106.0, 105.3, 70.6, 56.0, 55.2, 37.1.

#### 7-(3,5-Dimethoxy-benzyloxy)-6-methoxy-2-methylisoquinolin-1(2H)-one (14c)

The crude product was purified by flash column chromatography on silica gel yielding **14c** (75%) as a white solid. ESI-MS: 356.02 (M+1). HR-TOF-MS: calculated for C_20_H_21_NO_5_, 356.1498 (M+1); found 356.1502 (M+1). ^1^H-NMR (400 MHz, CDCl_3_): δ 7.86 (1H, s), 6.98 (1H, d, *J* = 7.6 Hz), 6.88 (1H, s), 6.65 (2H, s), 6.40 (1H, s), 6.39 (1H, d, *J* = 7.2 Hz), 5.20 (2H, s), 3.97 (3H, s), 3.79 (6H, s), 3.57 (3H, s). ^13^C-NMR (75 MHz, CDCl_3_): δ 160.8, 153.3, 148.1, 147.9, 138.6, 132.6, 131.1, 119.9, 109.3, 106.1, 105.4, 105.2, 100.0, 70.2, 56.0, 55.4 (2), 37.1.

#### 6-Methoxy-2-methyl-7-(4-methyl-benzyloxy)-isoquinolin-1(2H)-one (14d)

The crude product was purified by flash column chromatography on silica gel (MeOH∶CH_2_Cl_2_ = 3∶200) yielding **14d** (92%) as a white solid. mp: 150–152°C. ESI-MS: 310.12 (M+1). HR-TOF-MS: calculated for C_19_H_19_NO_3_, 310.1443 (M+1); found 310.1437 (M+1). ^1^H-NMR (400 MHz, CDCl_3_): δ 7.87 (1H, s), 7.38 (2H, d, *J* = 8.0 Hz), 7.18 (2H, d, *J* = 8.0 Hz), 6.97 (1H, d, *J* = 7.2 Hz), 6.86 (1H, s), 6.39 (1 H, d, *J* = 7.2 Hz), 5.22 (2H, s), 3.95 (3 H, s), 3.57 (3H, s), 3.17 (3H, s). ^13^C-NMR (100 MHz, CDCl_3_): δ 161.7, 153.5, 148.2, 137.6, 133.2, 132.5, 130.9, 129.1, 127.6, 119.9, 109.2, 106.0, 105.4, 70.7, 56.0, 37.1, 21.2.

#### 5-Benzyloxyl-6-methoxy-2-methylisoquinolin-1(2H)-one (15a)

The crude product was purified by flash column chromatography on silica gel yielding **15a** (100%) as a white solid. mp: 125–127°C. ESI-MS: 296.23 (M+1), 318.13 (M+Na). HR-TOF-MS: calculated for C_18_H_17_NO_3_, 296.1287 (M+1); found 296.1294 (M+1). ^1^H-NMR (400 MHz, CDCl_3_): δ 8.20 (1H, d, *J* = 8.8 Hz), 7.35–7.47 (5H, m) 7.14 (1H, d, *J* = 8.8 Hz), 6.94 (1H, d, *J* = 7.0 Hz), 6.67 (1H, d, *J* = 7.0 Hz), 5.07 (2H, s), 3.97 (3H, s), 3.53 (3H, s). ^13^C-NMR (75 MHz, CDCl_3_): δ 162.2, 154.4, 140.7, 137.3, 132.7, 132.5, 128.4, 128.3, 128.1, 124.6, 120.4, 112.2, 100.4, 75.3, 56.0, 36.8.

#### 6-Methoxy-5-(3-methoxy-benzyloxy)-2-methyl-2H-isoquinolin-1-one (15b)

The crude product was purified by flash column chromatography on silica gel (ethyl acetate∶hexane = 7∶3) yielding **15b** (100%) as a white solid. mp: 124–125°C. ESI-MS: 326.11 (M+1). HR-TOF-MS: calculated for C_19_H_19_NO_4_, 326.1392 (M+1); found 326.1408 (M+1). ^1^H-NMR (300 MHz, CDCl_3_): δ 8.20 (1H, d, *J* = 8.9 Hz), 7.29 (1H, t, *J* = 8.1 Hz), 7.14 (1H, d, *J* = 8.9 Hz), 7.02–7.05 (2H, m), 6.96 (1H, d, *J* = 7.5 Hz), 6.86–6.89 (1H, m), 6.69 (1H, d, *J* = 7.6 Hz), 5.05 (2H, s), 3.98 (3H, s), 3.82 (3H, s), 3.55 (3H, s). ^13^C-NMR (75 MHz, CDCl_3_): δ 162.2, 159.6, 154.4, 140.8, 138.9, 132.7, 132.5, 129.4, 124.6, 120.5, 120.4, 113.6, 112.2, 100.4, 75.1, 56.0, 55.2, 36.8.

#### 5-(3,5-Dimethoxy-benzyloxy)-6-methoxy-2-methylisoquinolin-1(2H)-one (15c)

The crude product was purified by flash column chromatography on silica gel (MeOH∶CH_2_Cl_2_ = 1∶99) yielding **15c** (100%) mp: 85–86°C. ESI-MS: 355.98 (M+1). HR-TOF-MS: calculated for C_20_H_21_NO_5_, 356.1498 (M+1); found 356.1499 (M+1). ^1^H-NMR (300 MHz, CDCl_3_): δ 8.20 (1H, d, *J* = 9.1 Hz), 7.14 (1H, d, *J* = 8.8 Hz), 6.96 (1H, d, *J* = 7.6 Hz), 6.70 (1H, d, *J* = 7.0 Hz), 6.64 (2H, s), 6.43 (1H, d, *J* = 2.1 Hz), 5.01 (2H, s), 3.99 (3H, s), 3.80 (6H, s), 3.55 (3H, s). ^13^C-NMR (75 MHz, CDCl_3_): δ 162.0, 160.7, 154.2, 140.7, 139.6, 132.6, 132.5, 124.6, 120.5, 112.2, 105.9, 100.4, 100.0, 75.2, 56.1, 55.4, 36.9.

#### 6-Methoxy-2-methyl-5-(4-methyl-benzyloxy)-isoquinolin-1(2H)-one (15d)

The crude product was purified by flash column chromatography on silica gel yielding **15d** (95%). ESI-MS: 310.11 (M+1). HR-TOF-MS: calculated for C_19_H_19_NO_3_, 310.1443 (M+1); found 310.1453 (M+1). ^1^H-NMR (400 MHz, CDCl_3_): δ 89.19 (1H, d, *J* = 8.8 Hz), 7.34 (1H, d, *J* = 7.6 Hz), 7.17 (2H, d, *J* = 7.6 Hz), 7.13 (1H, d, *J* = 9.2 Hz), 6.93 (1H, d, *J* = 7.6 Hz), 6.67 (1H, d, *J* = 7.6 Hz), 2.02 (2H, s), 3.97 (3H, s), 3.53 (3H, s), 2.35 (3H, s). ^13^C-NMR (100 MHz, CDCl_3_): δ 162.0, 154.2, 140.7, 137.7, 134.2, 132.6, 132.3, 128.9, 128.3, 124.3, 120.3, 112.2, 100.5, 75.2, 56.0, 36.8, 21.3.

### Biology

#### Stable cell lines

Individual melatonin receptor subtype (either MT_1_ or MT_2_) was stably co-expressed with a G protein chimera 16z25 in Chinese hamster ovary (CHO) cells, hereafter denoted as CHO-hMT_1_ and CHO-hMT_2_, respectively. The generation and characterization of the two stable cell lines have been described previously [39]. Single cell-derived colonies of the stable lines with sufficiently high receptor density and robust responses in the fluorometric assay (as described below) were isolated from the pool of transfected cell. Usage of these clonal lines was limited to 15 passages to prevent degradation of signal detection.

#### Competitive binding assay

Competitive binding assays were performed as described [26]. Cells were suspended in binding buffer (50 mM Tris, 2 mM MgCl_2_, 1 mM EGTA, pH 7.4) in the density of 1.5×10^5^ cell/ml. 1 nM [^3^H]melatonin and increasing concentrations of a tested compound was included. Cells were incubated at 4°C for 60 min with intermittent agitation and the reaction was stopped by rapid filtration through GF/C filters pre-soaked in 10 mM Tris, pH 7.4 and washed with 1 ml of prechilled binding buffer. Retained radioactivity was measured with Wallac 1450 Microbeta Jet scintillation counter. Competition curves were drawn as one-site competition nonlinear regression model using GraphPad Prism 3.03 to the data which were means ± SEM of 3–4 independent experiments performed in duplicates. In every assay, melatonin was included as standard reference with highly reproducible K_i_ values, which were calculated using the Cheng-Prusoff equation.

#### Fluorescence-based assay of intracellular Ca2+ mobilization using FLIPR^TETRA^

4×10^4^ Cells were seeded and grown for overnight in Costar #3603 96-well plates before assay. The growth medium was replaced by 200 µl of 2 µl Fluo-4 AM (Invitrogen) in the assay buffer (Ca^2+^-containing HEPES-buffered HBSS with 2.5 mM probenecid) to label the cells for 1 h at 37°C. Tested compounds in different concentrations were diluted in assay buffer in 5× of the final desired concentrations in a V-bottomed microplate, and both the cell and compound plates were put into the fluorometric imaging plate reader FLIPR^TETRA^ (Molecular Devices, Sunnyvale, CA). Upon the addition of tested compounds or melatonin, the fluorescent signal was monitored real-time for 3 min with an excitation wavelength of 488 nm as described previously [25]. The peak of the time course of fluorescence changes was expressed in relative fluorescence units (RFU). Dose response curves were constructed by non-linear regression using GraphPad Prism 3.

#### cAMP accumulation assay

2×10^5^ cells were seeded in each well of 12-well plates and grown for overnight. Each well was labeled with 1 ml of [^3^H]adenine (1 µCi/ml) in F-12K medium containing 1% FBS (vol/vol) for overnight. The labeling media were then replaced by 1 ml of 20 mM HEPES-buffered F-12K containing 1 mM isobutylmethylxanthine (IBMX) and the tested compounds at desired concentrations, and incubated at 37°C for 30 min. The reaction was stopped by aspiration and adding 1 ml prechilled 5% trichloroacetic acid (w/v) with 1 mM ATP, and stored at 4°C for 30 min. [^3^H]cAMP was extracted from the pool of labeled adenosine nucleotides by sequential ion-exchange chromatography as previously described [40]. The radioactivity of labeled cAMP and total labeled nucleotide fractions separated by chromatography were estimated by scintillation counting. Inhibition curves were constructed by non-linear regression using GraphPad Prism 3.

#### Immunodetection of ERK phosphorylation

2×10^5^ cells were seeded into each well of 12-well plates for overnight before serum withdrawal for 4 h to reduce the basal ERK phosphorylation. Tested compounds were diluted in serum-free culture medium at desired concentrations and treated the cells for 5 min. Reactions was stopped by aspiration and then adding 150 µl of lysis buffer with protease inhibitors (50 mM Tris–HCl, pH 7.5, 100 mM NaCl, 5 mM EDTA, 40 mM NaP_2_O_7_, 1% Triton X-100, 1 mM dithiothreitol, 200 µM Na_3_VO_4_, 100 µM phenylmethylsulfonyl fluoride, 2 µg/ml leupeptin, 4 µg/ml aprotinin, and 0.7 µg/ml pepstain). Cells were allowed to lyse for 30 min at 4°C with agitation, and the collected total cell lysates were cleared by centrifugation. Protein samples were resolved in SDS-PAGE and transferred to nitrocellulose membranes using iBlot system (Invitrogen). Phosphorylated ERK was detected using specific antibodies as described previously [41]. Chemiluminescence signals on the blots were detected on X-ray films.

## Supporting Information

Figure S1 **Phosphorylation of ERK induced by compounds 6 and 12.** Experimental details were as to the legend of Figure 5.
(TIF)

Figure S2 **Regulation of intracellular Ca^2+^ mobilization in CHO cells expressing MT_1_ or MT_2_ by 7e or luzindole.** Experimental details were as to the legend of Figure 2. Estimation of maximal responses and EC_50_ were tabulated in Table 2.
(TIF)

Figure S3 **Comparison of ERK phosphorylation inhibition by** ***para*** **-substituted benzyloxyl derivatives.** Experimental details were as to the legend of Figure 7.
(TIF)
