# Supplementary figures and images for: Synthesis and Functional Characterization of Substituted Isoquinolinones as MT2-Selective Melatoninergic Ligands
Source: PLoS One. 2014 Dec 5;9(12):e113638. doi: 10.1371/journal.pone.0113638 (PMC4257560; doi:10.1371/journal.pone.0113638)

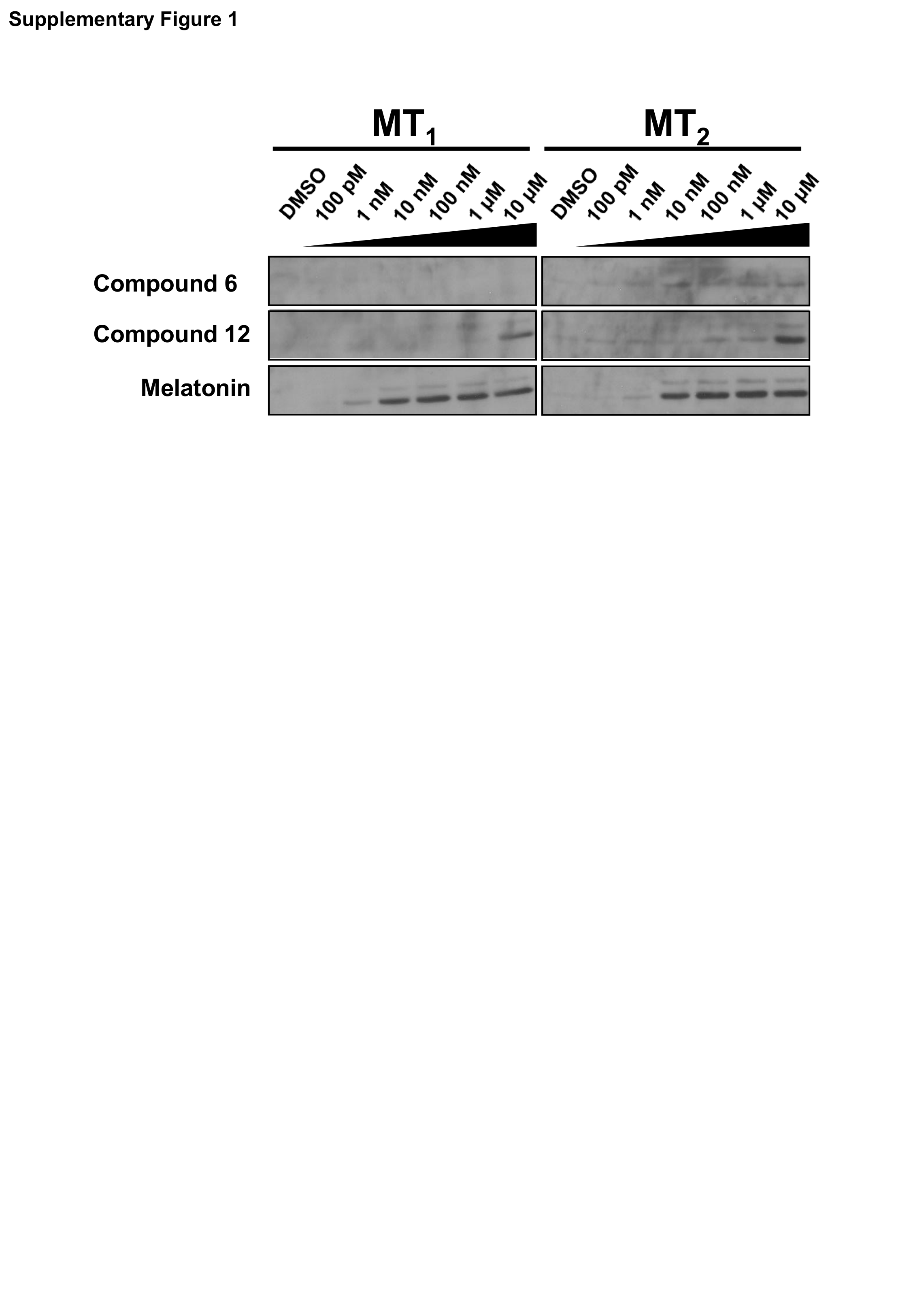

Supplement: Figure S1 — Phosphorylation of ERK induced by compounds 6 and 12. Experimental details were as to the legend of Figure 5. (TIF) [file pone.0113638.s001.tif]

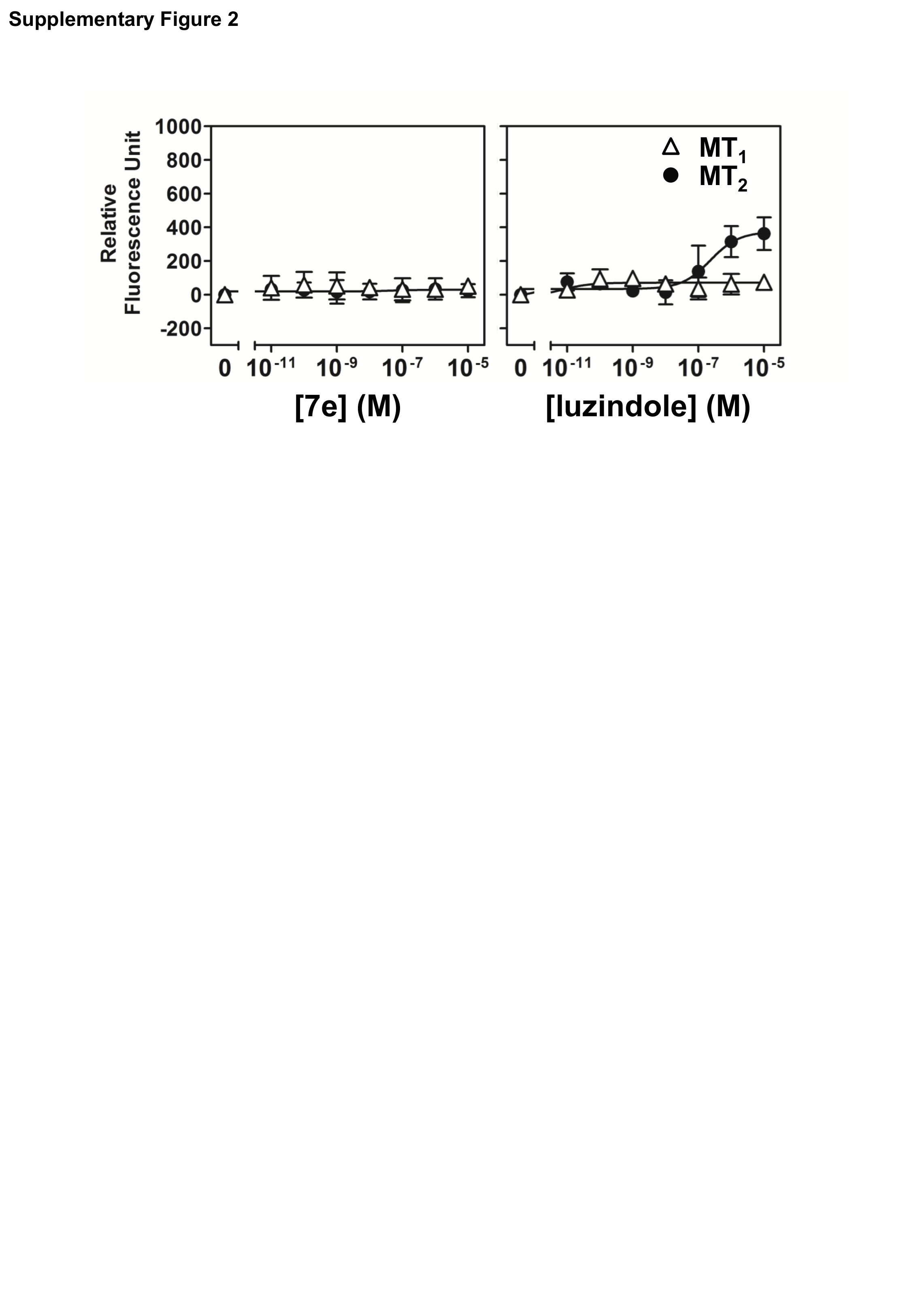

Supplement: Figure S2 — Regulation of intracellular Ca2+ mobilization in CHO cells expressing MT1 or MT2 by 7e or luzindole. Experimental details were as to the legend of Figure 2. Estimation of maximal responses and EC50 were tabulated in Table 2. (TIF) [file pone.0113638.s002.tif]

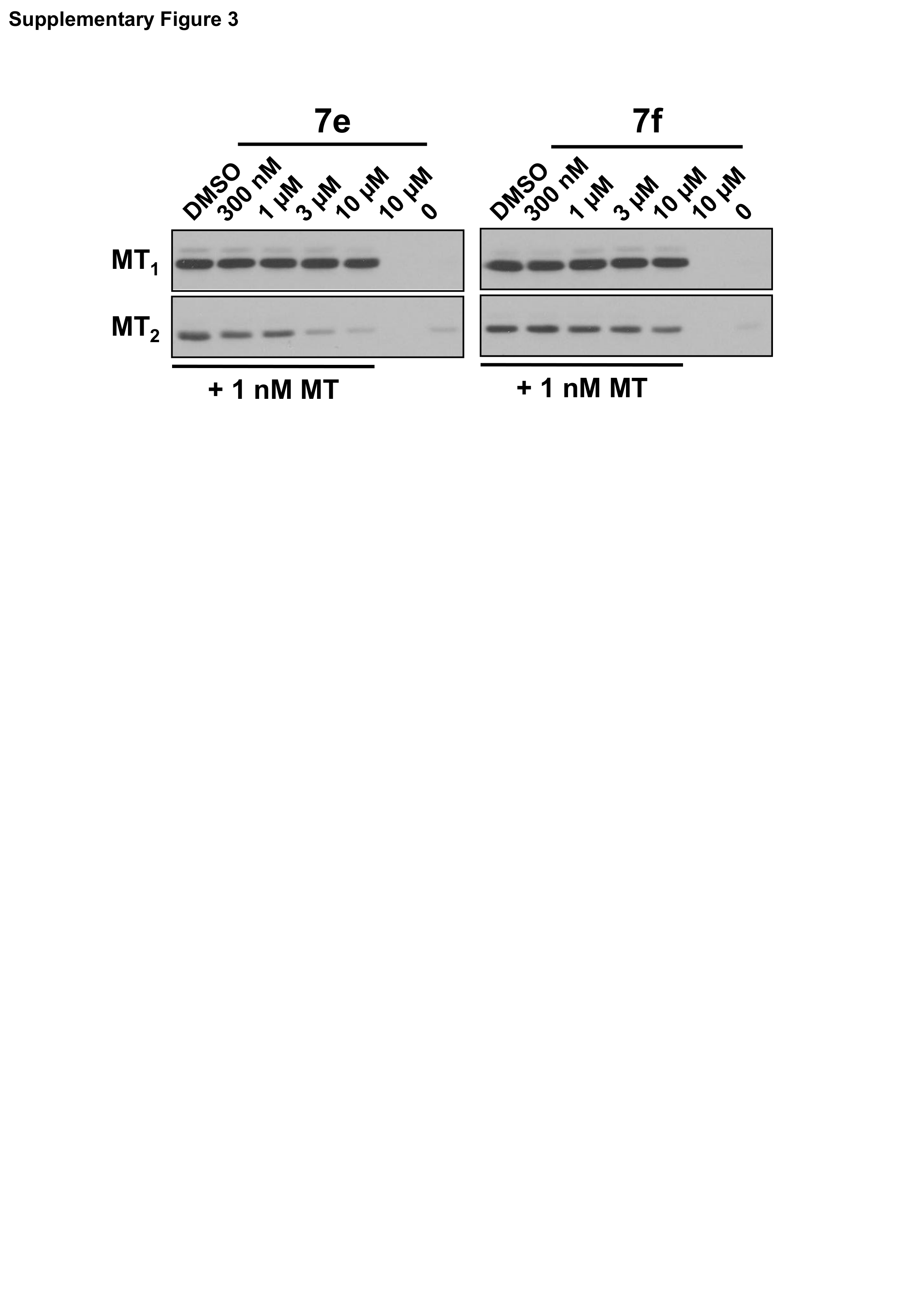

Supplement: Figure S3 — Comparison of ERK phosphorylation inhibition by para -substituted benzyloxyl derivatives. Experimental details were as to the legend of Figure 7. (TIF) [file pone.0113638.s003.tif]
